# Mechanistic Actions between *Garcinia atroviridis* Essential Oil and 2 Deoxy-d-glucose in Cultured PANC-1 Human Pancreatic Cancer Cells

**DOI:** 10.3390/molecules26123518

**Published:** 2021-06-09

**Authors:** Nik Nur Syazni Nik Mohamed Kamal, Fatin Athirah Abdul Aziz, Wen-Nee Tan, Agustine Nengsih Fauzi, Vuanghao Lim

**Affiliations:** 1 Integrative Medicine Cluster, Advanced Medical and Dental Institute, Universiti Sains Malaysia, Bertam 13200, Kepala Batas, Penang, Malaysia; fatinathirah938@gmail.com (F.A.A.A.); vlim@usm.my (V.L.); 2 Chemistry Section, School of Distance Education, Universsiti Sains Malaysia, Gelugor 11800, Penang, Malaysia; twn@usm.my; 3 Department of Chemical Pathology, School of Medical Sciences, Universiti Sains Malaysia, Kubang Kerian 16150, Kelantan, Malaysia; agustine@usm.my

**Keywords:** essential oil, 2-deoxy-d-glucose, PANC-1 cells, anti-proliferative, synergism

## Abstract

Pancreatic cancer is an aggressive disease that progresses in a relatively symptom-free manner; thus, is difficult to detect and treat. Essential oil is reported to exhibit pharmacological properties, besides its common and well-known function as aromatherapy. Therefore, this study herein aimed to investigate the anti-proliferative effect of essential oil extracted from leaves of *Garcinia atroviridis* (EO-L) against PANC-1 human pancreatic cancer cell line. The cell growth inhibitory concentration at 50% (IC_50_) and selective index (SI) values of EO-L analyses were determined as 78 µg/mL and 1.23, respectively. Combination index (CI) analysis revealed moderate synergism (CI values of 0.36 to 0.75) between EO-L and 2 deoxy-d-glucose (2-DG) treatments. The treatments of PANC-1 cells with EO-L, 2-DG and EOL+2DG showed evidence of depolarization of mitochondrial membrane potential, cell growth arrest and apoptosis. The molecular mechanism causing the anti-proliferative effect between EO-L and 2-DG is potentially through pronounced up-regulation of *P53* (4.40-fold), *HIF1α* (1.92-fold), *HK2* (2.88-fold) and down-regulation of *CYP3A5* (0.11-fold), as supported by quantitative mRNA expression analysis. Collectively, the current data suggest that the combination of two anti-proliferative agents, EO-L and 2-DG, can potentially be explored as therapeutic treatments and as potentiating agents to conventional therapy against human pancreatic cancer.

## 1. Introduction

According to Global Cancer Statistics 2018 [1], cancer was the leading cause of death worldwide prior to the impact of the coronavirus pandemic (COVID-19). Among the non-communicable diseases cluster, pancreatic cancer is shown to be the sixth leading cause of global cancer death [2]. Pancreatic cancer is an aggressive disease that develops in a relatively symptom-free manner and is commonly metastasized at the time of diagnosis as unresectable tumors [3,4]. Hence, for all the combined pancreatic cancer stages, the 1-year survival rate is around 20%, while the overall 5-year survival rate is less than 5% [5]. Pancreatic cancer is usually treated by complete surgical resection that slightly increases the 5-year survival chances to between 15 and 20% in patients [5]. Besides surgery or radiation procedures, patients are also given gemcitabine as a preferred first-line chemotherapy treatment [3,6]. However, pancreatic cancer may develop a high level of intrinsic or acquired drug resistance, thus reducing the effectiveness of gemcitabine treatment. As reported by Min et al., the median survival time of patients treated with gemcitabine is only 6.3 months [7]. Taken together, the aforementioned demography demonstrated that the mortality rate for pancreatic cancer remains high, possibly due to poor prognosis, limited diagnostics and poor responsiveness towards therapeutic modalities. Pancreatic ductal is the most common and fatal form of pancreatic cancer [8]. About 90% of pancreatic cancers, also known as pancreatic ductal adenocarcinoma (PDAC) [6], are adenocarcinoma and originate from the exocrine part of the pancreas. In this study, the PANC-1 cell line was used to represent the primary tumor of PDAC. In culture conditions, the doubling times are 28 h for PANC-1 cells [4]. 

Phytochemical constituents or bioactive metabolites of medicinal plants usually exhibit different profiles of pharmacological effects. In general, these phytochemical constituents can be categorized as primary and secondary metabolites based on their role in basic metabolic processes [9]. As mentioned by Hussein and El-Anssary [9], primary plant metabolites, namely amino acids, organic acids or nucleosides, are involved in essential life functions, such as cell division and growth, respiration, storage and reproduction. These metabolites are known to be involved in biological processes, such as glycolysis, the Krebs or citric acid cycle, photosynthesis and associated pathways [10]. On the other hand, plant secondary metabolites are the end-product in plant biosynthetic pathways [11]. Several important organoleptic characteristics, such as aroma, color and fruit nutritional value, could be contributed by the secondary metabolite contents [12]. Secondary metabolites perform their function of molecule signaling to protect the plants from biotic and abiotic stresses [12]. These metabolites have been evidenced to be used in traditional medicine for hundreds of years, as they demonstrate considerable biological activities. Basically, medicinal and aromatic plants generate a wide range of secondary metabolites such as terpenoids, alcoholic compounds, aldehydes, ketonic bodies and phenols [13]. Among those, terpenes, terpenoids and aromatic phenols are found to have major roles in the composition of various essential oils (EO) [13]. EOs are concentrated hydrophobic fractions commonly found in aromatic plants. EOs can be extracted from various plant parts such as twigs, flowers, leaves, bark, seeds and roots. The EO of aromatic and medicinal plants is reported to be effective against insecticidal activity [14], and it promotes antioxidant [15] and antimicrobial [16] activities. For example, the EOs of *Tanacetum nubigenum* demonstrate potent repellent and fumigant toxicities against *Tribolium castaneum*, a pest that affects wheat during storage [17]. EO from *Garcinia celebica* L. has antimicrobial activity against *Bacillus subtilis*, Methicillin-resistant *Staphylococcus aureus* and *Proteus mirabilis*, and it thus effective against both Gram-positive and Gram-negative bacteria [16]. EO obtained from the stem bark of *Garcinia atroviridis* was found to be effective in preventing the destructive process caused by oxidative stress through the promotion of antioxidant activity [18]. Due to these promising therapeutic properties, EO and its components have been used for a wide range of applications in the pharmaceutical, cosmetic and food industries [13]. 

*Garcinia* clan plants are distributed widely in Asian tropical regions. One of the example species is *Garcinia atroviridis* (*Asam Gelugur*). It is a fruit tree commonly found in forests of Peninsular Malaysia [19]. It shares a similar family to Mangosteen, which belongs to the Guttiferales ordo and Guttiferae family [19]. It can grow to a height of 20 m, have red-yellow flowers with four free sepals and four free petals, deep-green glossy leaves and fruits with an orange-yellow color [20,21]. The fruits and leaves of this plant have an aromatic smell. This aromatic medicinal plant generates EO in the form of secondary metabolites. Recently, major secondary metabolites of essential oils were successfully extracted from the leaves and stem bark of *G. atroviridis* [22]. The oils exhibited potent cytotoxicity activity against MCF-7 human breast cancer cells with inhibitory concentration at a 50% (IC_50_) value of 71 µg/mL. However, the inhibitory action of essential oils extracted from *G. atroviridis* on pancreatic carcinogenesis has never been explored. Therefore, the aim of this paper was to investigate the mechanistic actions possessed by EO obtained from leaves of *G. atroviridis* from Penang, Malaysia, against PANC-1 human pancreatic cells. 

## 2. Results

### 2.1. Antiproliferative Effects of EO-L and 2-DG

**Figure 1:**
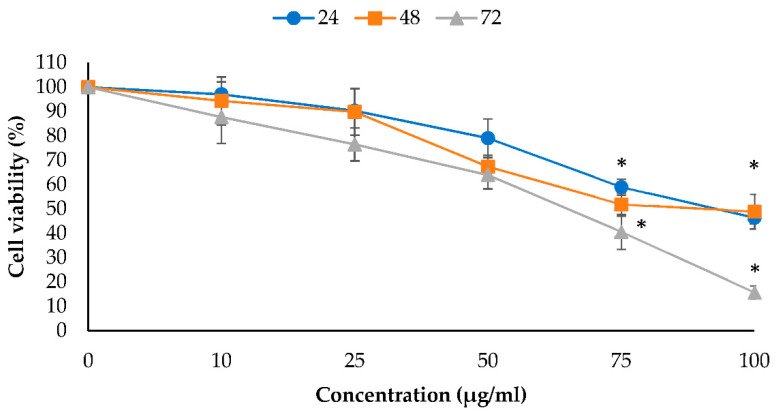
Anti-proliferative effect of EO-L in PANC-1 cells. PANC-1 cells were treated with 10–100 µg/mL of EO-L for 24, 48 and 72 h. The anti-proliferative effect was measured by MTT assay. The data were shown as the mean values ± S.D. for three independent experiments, and the statistical analysis was determined using a Student’s *t*-test, with the * symbol showing a significant difference (*p* < 0.05) in comparison to control untreated cells.

The anti-proliferative effects of EO-L and 2-DG were determined by using an MTT assay, as described in Section 4.3. EO-L inhibited the growth of PANC-1 cells in a concentration- and time-dependent manner (Figure 1). EO-L at concentrations of 75 and 100 µg/mL resulted in a significant anti-proliferative effect on the PANC-1 cells throughout all the incubation periods tested. The EO-L concentration of 100 µg/mL decreased the viability of PANC-1 cells to 46.3% (*p* < 0.05), 48.9% (*p* < 0.05) and 15.7% (*p* < 0.05) after 24, 48 and 72 h of treatment, respectively.

**Figure 2:**
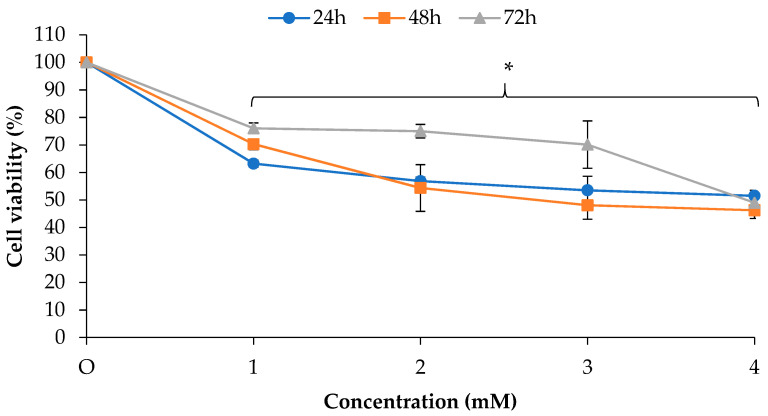
Anti-proliferative effect of 2-DG in PANC-1 cells. PANC-1 cells were treated with 1–4 mM of 2-DG for 24, 48 and 72 h. The anti-proliferative effect was measured by MTT assay. The data were shown as the mean values ± S.D. for three independent experiments, and the statistical analysis was determined using a Student’s *t*-test, with the * symbol showing a significant difference (*p* < 0.05) in comparison to control untreated cells.

Figure 2 shows the anti-proliferative effect of 2-DG in PANC-1 cells. Essentially, 2-DG is a synthetic glucose analogue that has shown the capability to inhibit glucose metabolism and ATP production [23]. At 24 h post treatment, 1 and 4 mM of 2-DG were observed to decrease the viability of PANC-1 cells to 63.2% (*p* < 0.05) and 51.5% (*p* < 0.05), respectively. Continuous 48 and 72 h incubation with 4 mM of 2-DG showed stronger anti-proliferative effects, with 46.2% (*p* < 0.05) and 48.9% (*p* < 0.05) remaining viable cells being measured, respectively. 

### 2.2. Cytotoxic Effects of EO-L and 2-DG

**Figure 3:**
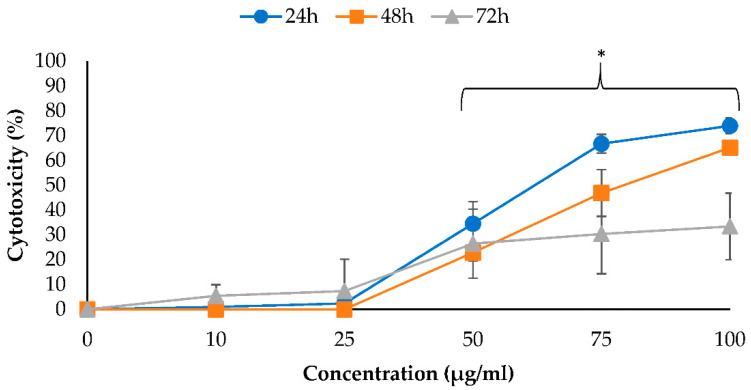
The cytotoxic effect of EO-L (10–100 µg/mL) was assessed by LDH assay. Treatments were performed for 24, 48 and 72 h, and results are expressed as percentage of cytotoxicity at various concentrations. The data were shown as the mean values ± S.D. for three independent experiments, and the statistical analysis was determined using a Students *t*-test, with the * symbol showing a significant difference (*p* < 0.05) in comparison to control untreated cells.

This study also evaluated the cytotoxic effects of EO-L and 2-DG against PANC-1 cells by determining the release of lactate dehydrogenase (LDH) into the cell culture medium, as described in Section 4.4. This cytosolic enzyme is known to be released by damaged cells only. Therefore, it is commonly used as a biomarker for cytotoxicity determination of an agent. Figure 3 shows the percentage of LDH released from PANC-1 cells upon 24–72 h treatment with EO-L. The treatment of PANC-1 cells with increasing concentrations of EO-L (10–100 µg/mL) produced an exponential increase in LDH, and the enzymes were released as early as 24 h. The percentages of LDH released were calculated as 34.49% (*p* < 0.05), 66.76% (*p* < 0.05) and 73.97% (*p* < 0.05) for 50, 75 and 100 µg/mL of EO-L concentrations, respectively. The percentage of LDH released was decreased following 48 h and 72 h incubation periods, as the cells were killed after initial treatment, with little or no cell division occurring following a prolonged incubation period.

**Figure 4:**
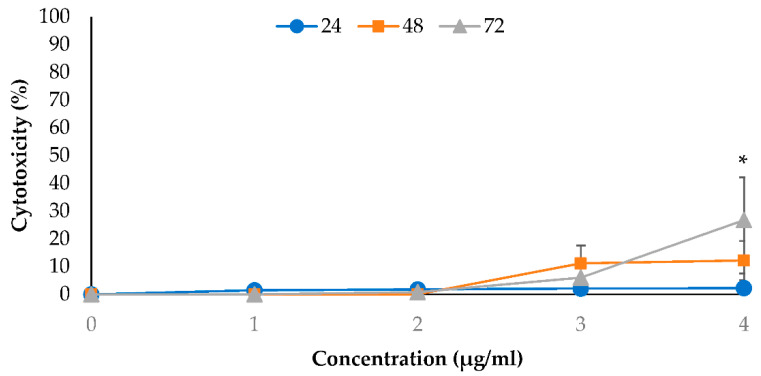
The cytotoxic effect of 2-DG (1–4 mM) was assessed by LDH assay. Treatments were performed for 24, 48 and 72 h and results are expressed as percentage of cytotoxicity at various concentrations. The data were shown as the mean values ± S.D. for three independent experiments, and the statistical analysis was determined using a Student’s *t*-test, with the * symbol showing a significant difference (*p* < 0.05) in comparison to control untreated cells.

Figure 4 shows the cytotoxic effect of 2-DG against PANC-1 cells. This study found that 2-DG at 1, 2 and 3 mM did not exhibit any appreciable effects on the LDH released against PANC-1 cells within 24 to 72 h post-treatments. Progressive cytotoxicity only occurred at 4 mM 2-DG concentration as the exposure time was increased from 24 to 72 h. At this concentration, the percent of cytotoxicity was approximately 2.24%, 12.2% and 26.7% (*p* < 0.05) after 24, 48 and 72 h, respectively.

### 2.3. Inhibitory Concentration at 50% (IC_50_) and Selectivity Index (SI) Values of EO-L

**Figure 5:**
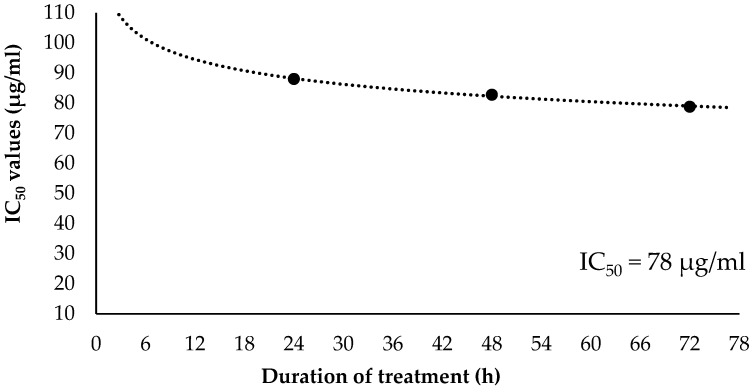
Determination of constant IC_50_ value of EO-L in PANC-1 cell line.

Figure 5 shows the curve plotted with the IC_50_ values of EO-L treatment on PANC-1 cells against the duration of treatment. Each IC_50_ value produced at specific incubation period was plotted, and the curve was drawn until it reached a constant asymptote. In PANC-1 cells, the IC_50_ values obtained at 24 h, 48 h and 72 h were 88 µg/mL, 82 µg/mL and 78 µg/mL, respectively. The plot in Figure 5 indicates that the IC_50_ values of EO-L gradually decrease with a prolonged incubation period in PANC-1 cells. As indicated in Figure 5, the constant IC_50_ value of EO-L in PANC-1 cells was established to be 78 µg/mL.

**Figure 6:**
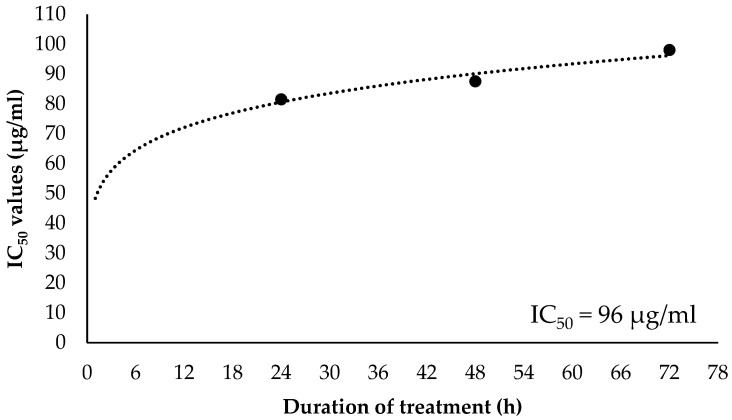
Determination of constant IC_50_ value of EO-L in BEAS-2B cell line.

The constant IC_50_ value of EO-L was also determined in BEAS-2B non-cancerous cell line. In these cells, IC_50_ values obtained at 24 h, 48 h and 72 h were 80 µg/mL, 90 µg/mL and 96 µg/mL, respectively (Figure 6). The plot in Figure 6 indicates that the IC_50_ values of EO-L gradually increase with a prolonged incubation period in BEAS-2B non-cancerous cells. The constant IC_50_ value of EO-L in BEAS-2B cells was determined to be 96 µg/mL.

The selectivity index (SI) value was calculated based on the ratio of IC_50_ value obtained in the BEAS-2B cell line compared to the PANC-1 cell line. Thus, the SI of EO-L at 24 h, 48 h and 72 h was measured as 0.9, 1.09 and 1.23, respectively.

### 2.4. Combination Index of EOL+2DG on PANC-1 Cell Line

**Figure 7:**
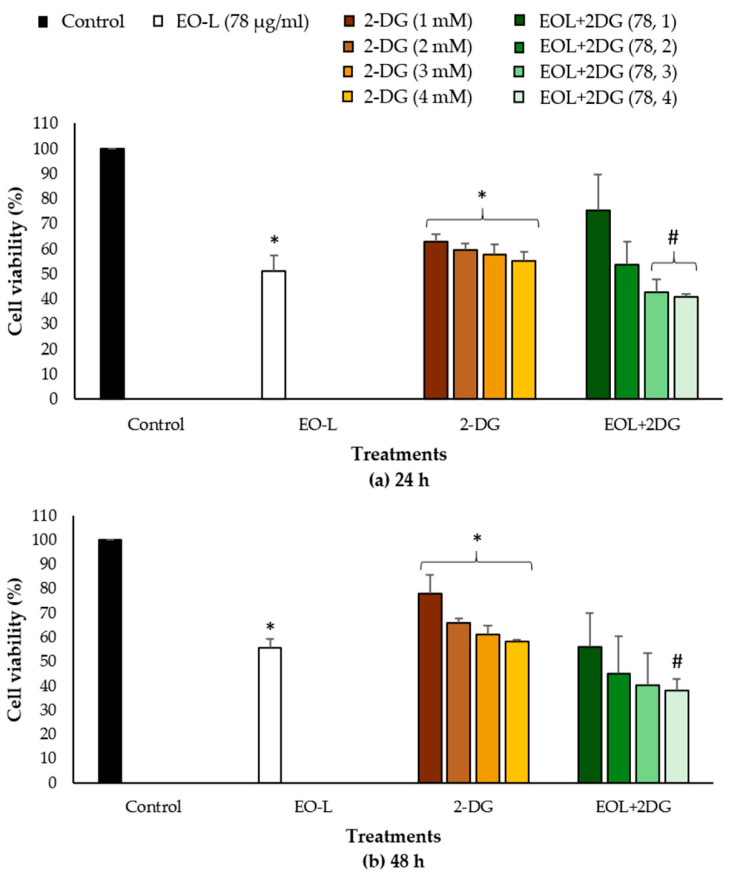

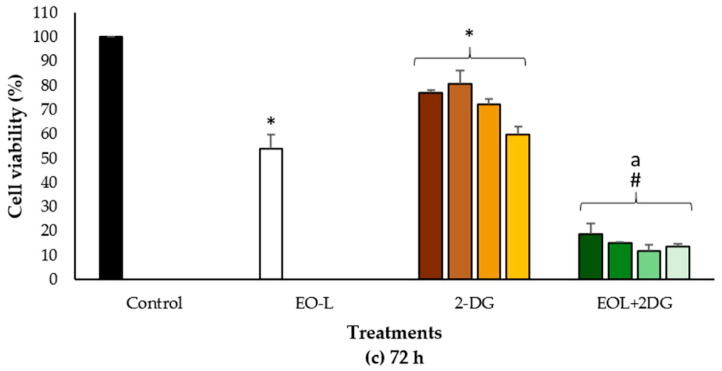
Anti-proliferative effects of EOL+2DG in comparison to untreated, EO-L and 2-DG treatments in PANC-1 cells at (**a**) 24 h, (**b**) 48 h and (**c**) 72 h time points. The data were shown as the mean values ± S.D. for three independent experiments, and the statistical analysis was determined using a Student’s *t*-test, with the * symbol showing a significant difference (*p* < 0.05) in comparison to control untreated cells, the # symbol showing a significance difference (*p* < 0.05) in comparison to 2-DG and the a symbol showing a significant difference (*p* < 0.05) in comparison to EO-L.

Figure 7a–c shows the percentages of anti-proliferative effects of EOL+2DG in comparison to EO-L (IC_50_) and 2-DG (1, 2, 3 and 4 mM), while culture medium alone was used as a vehicle control. In comparison to 2-DG treatment, the combination of EO-L and 2-DG at 3 and 4 mM concentrations was observed to significantly (*p* < 0.05) inhibit cell proliferation in PANC-1 cells. Similarly, the EOL+2DG combination exhibited stronger growth inhibition in PANC-1 cells than EO-L alone after 72 h of incubation (Figure 7). At this time point, the proliferation of PANC-1 cells was observed to decline to about 15 to 20% (*p* < 0.05) in all combination sets explored.

The percentages of anti-proliferative activities caused by combinational treatments were further analyzed using CompuSyn software version 1.0 to calculate the combination index (CI). The combination index (CI) provided a quantitative measure of the degree of drug interaction between, for example, EO-L and 2-DG. The values can be translated as synergism (CI < 1), additive effect (CI = 1) or antagonism (CI > 1) for a given endpoint of the effect measurements.

As shown in Table 1, all combinational sets between EO-L (IC_50_) and 2-DG concentrations exhibited synergism. The CI values were calculated between 0.3 and 0.7 (synergism) and 0.7 and 0.85 (moderate synergism), depending on the concentration of 2-DG and incubation period used in the treatments. The CI values of 0.35 ± 9.3 and 0.76 ± 4.8 were generated by the combinations with 2 mM and 4 mM concentrations of 2-DG, respectively. 

### 2.5. Effects of EO-L, 2-DG and EOL+2DG on Mitochondrial Membrane Potential (MMP) in PANC-1 Cells

**Figure 8:**
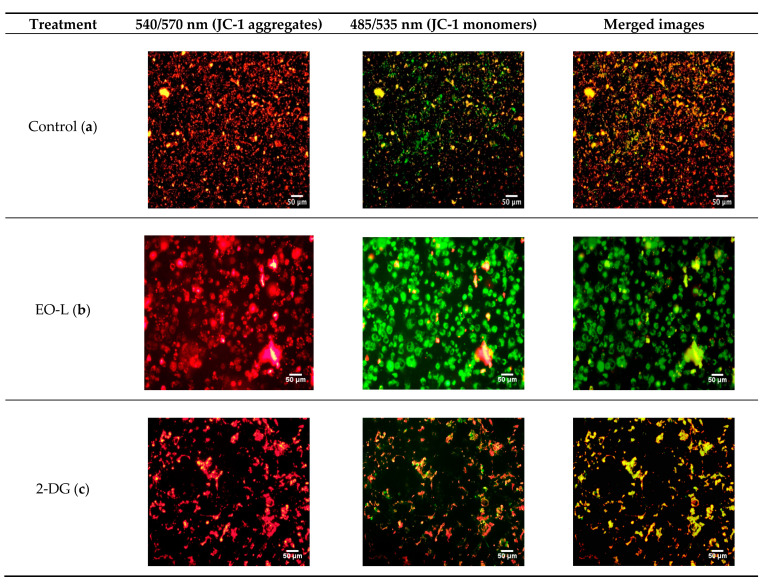

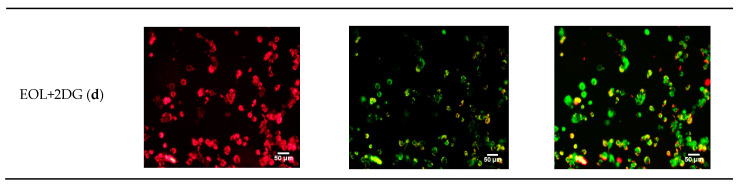
Fluorescence microscopy images of PANC-1 cells stained with JC-1 dye. Green fluorescence—depolarized (monomer) mitochondria; red fluorescence—(J-aggregates) mitochondria. PANC-1 cells were treated with (**a**) culture medium, (**b**) EO-L, (**c**) 2-DG and (**d**) EOL+2DG for 24 h.

In this study, a fluorescent dye (JC-1) was used to investigate the effects of EO-L, 2-DG and EOL+2DG in comparison to vehicle control (untreated cells). JC-1 dye emits from green to red as the mitochondrial membrane potential increases. Untreated cells were found with high JC-1 aggregates, and they emitted a strong red color (Figure 8). As shown in Figure 8c, a combination of red and green fluorescence was observed in the cells treated with 2-DG alone. This is an indication of a transition from polarized to depolarized membrane potential after 24 h of exposure to 2-DG. Fluorescence microscopic analysis demonstrated that both the EO-L and EOL+2DG induced mitochondrial depolarization in PANC-cells after 24 h of treatments, as depicted by the strong green fluorescence monomeric form of JC-1 (as shown in Figure 8b,d, respectively). These were indications of a prominent loss of membrane potential in PANC-1 pancreatic cancer cells that were treated with EO-L and EOL+2DG.

In this study, the mitochondrial membrane potential changes (Δ ψm) of PANC-1 cells were also evaluated quantitatively after 24, 48 and 72 h exposures to control, EO-L, 2-DG and EOL+2DG. The percentages of polarized and depolarized mitochondria cells were calculated using the following formula:

(∆ψm treated cell)/(∆ψm untreated cell) × 100(1)

**Figure 9:**
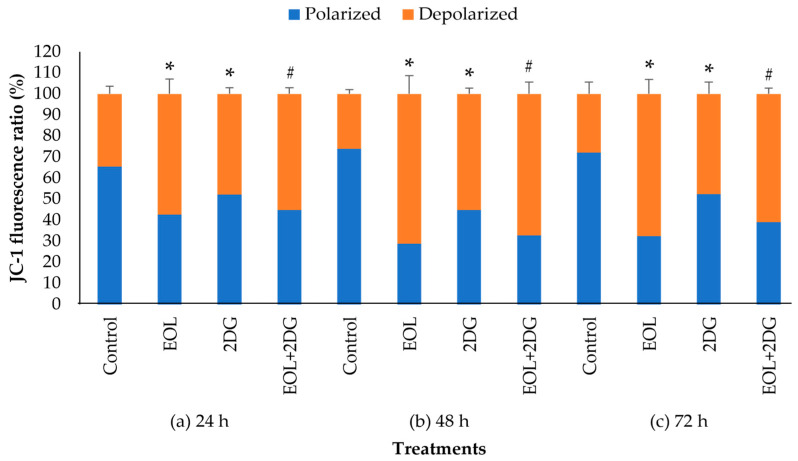
A quantitative analysis of mitochondrial membrane potential in PANC-1 cells: The percentages of polarized and depolarized cells after (**a**) 24 h, (**b**) 48 h and (**c**) 72 h exposures to culture medium (vehicle control), EO-L, 2-DG and EOL+2DG, respectively. The data were shown as the mean values ± S.D. for three independent experiments, and the statistical analysis of depolarized cells was determined using a Student’s *t*-test, with the * symbol showing a significant difference (*p* < 0.05) in comparison to control untreated cells and the # symbol showing a significant difference (*p* < 0.05) in comparison to 2-DG.

As shown in Figure 9, all the treatments cause a significant (*p* < 0.05) increased in the percentage of depolarized mitochondrial cells when compared to vehicle control. The percentages of cells with depolarized mitochondria acquired were 57.4%, 47.8% and 55.1%, after 24 h treatments with EO-L, 2-DG and EOL+2DG, respectively (Figure 9a). At 48 h, a higher percentage of depolarized mitochondria was observed in the cells treated with EO-L (71.2%) and EOL+2DG (67.2%) (Figure 9b). Similar observations were evidenced after 72 h of exposure to each treatment (Figure 9c). These results demonstrated that EO-L consistently induced mitochondrial depolarization in PANC-1 cells. Furthermore, the level of cells with depolarized mitochondria was higher in EOL+2DG in comparison to 2-DG alone. 

### 2.6. Effects of EO-L, 2-DG and EOL+2DG on Mechanisms of Cell Death (Apoptosis and/or Necrosis) in PANC-1 Cells

**Figure 10:**
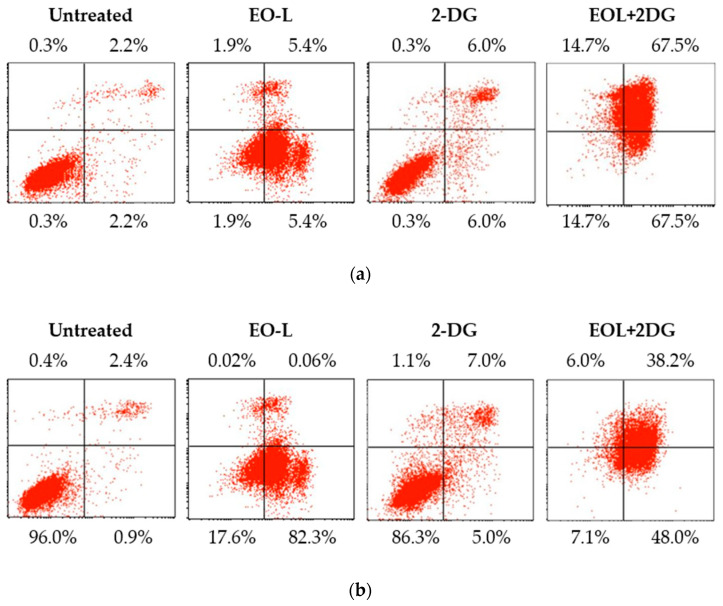

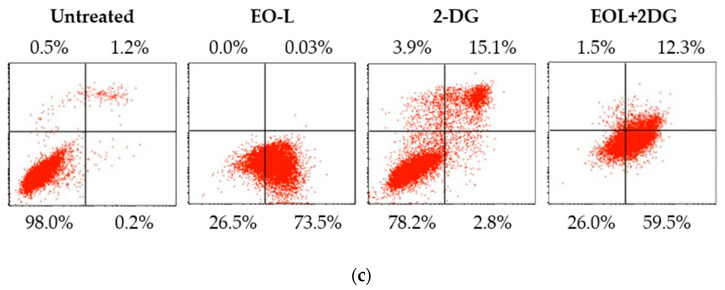
Representative cytogram of PANC-1 cells: PANC-1 cells were exposed to untreated (culture medium), EO-L, 2-DG and EOL+2DG for (**a**) 24 h, (**b**) 48 h and (**c**) 72 h. Quadrant location for the representative dot plots: lower left—FITC^−^/PI^−^ (viable cells); lower right—FITC^+^/PI^−^ (early apoptotic cells); upper right—FITC^+^/PI^+^ (late apoptotic cells); and upper left—FITC^−^/PI^+^ (necrotic cells).

Figure 10 shows a representative cytogram acquired from flow cytometry after 24, 48 and 72 h post-treatment. As shown in Figure 10a, the EO-L treatment caused an accumulation of PANC-1 cells in the lower right quadrant (early apoptosis) after 24 h of treatment. On the contrary, the 2-DG treatment caused the cells to be mostly accumulated in the lower left quadrant (viable). The EOL+2DG treatment resulted in a marked accumulation of cells in the upper right quadrant, indicating a late stage of apoptosis compared to control, EO-L and 2-DG. A progressive increase in the accumulation of early apoptotic and late apoptotic cells was observed in the cells treated with EO-L and EOL+2DG, respectively (as shown in Figure 10b,c). However, the treatments of PANC-1 cells with 2-DG for 48 and 72 h did not result in a greater number of cell deaths, apoptosis or necrosis.

**Figure 11:**
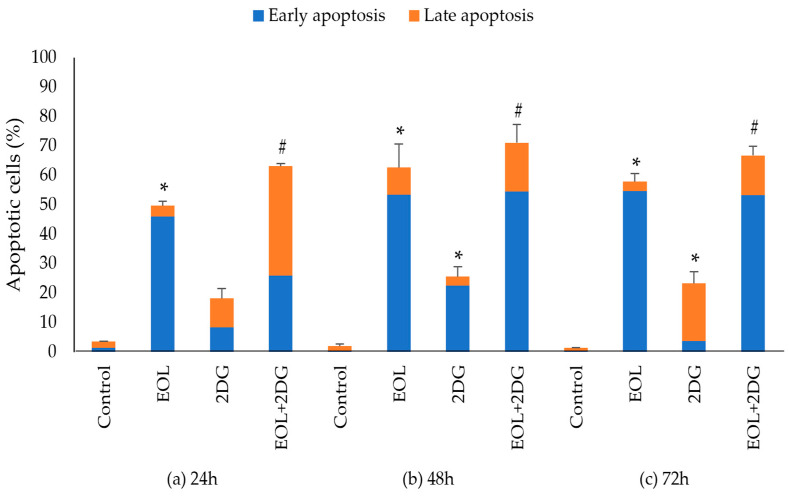
Induction of apoptosis in PANC-1 cells at (**a**) 24 h, (**b**) 48 h and (**c**) 72 h. The data were shown as the mean values ± S.D. for three independent experiments, and the statistical analysis was determined using a Student’s *t*-test, with the * symbol showing a significant difference (*p* < 0.05) in comparison to control untreated cells and the # symbol showing a significant difference (*p* < 0.05) in comparison to 2-DG.

The percentage of total apoptotic cells is referred to the sum of both early and late apoptotic percentage values obtained from three biological repeats (Figure 11). At 24 h, the percentages of total apoptotic cells induced by EO-L, 2-DG and EOL+2DG were measured at 49.7%, 18.2% and 63.1%, respectively (Figure 11a). Flow cytometric analysis showed that the combination of EO-L with 2-DG resulted in the promotion of apoptosis, with a significant increase in late-stage apoptosis of PANC-1 cells compared to either treatment alone (*p* < 0.05). 

At 48 h, the percentages of total apoptosis were observed to increase from 62.7%, 25.6% and 71.1% after the cells were treated with EO-L, 2-DG and EOL+2DG, respectively (Figure 11b). Similar results were also obtained 72 h post-treatment, with 57.91%, 23.3% and 66.78% of total apoptosis for EO-L, 2-DG and EOL+2DG (Figure 11c). Both EO-L and EOL+2DG consistently induced apoptosis in PANC-1 cells throughout the three incubation periods tested in this study. Besides, the percentages of late apoptosis increased significantly with the co-administration of EO-L compared to 2-DG alone. These observations indicated that EO-L promotes apoptotic cell death and also enhances the level of apoptosis.

### 2.7. Effects of EO-L, 2-DG and EOL+2DG on Mechanisms of Cell Cycle Distribution in PANC-1 Cells

**Figure 12:**
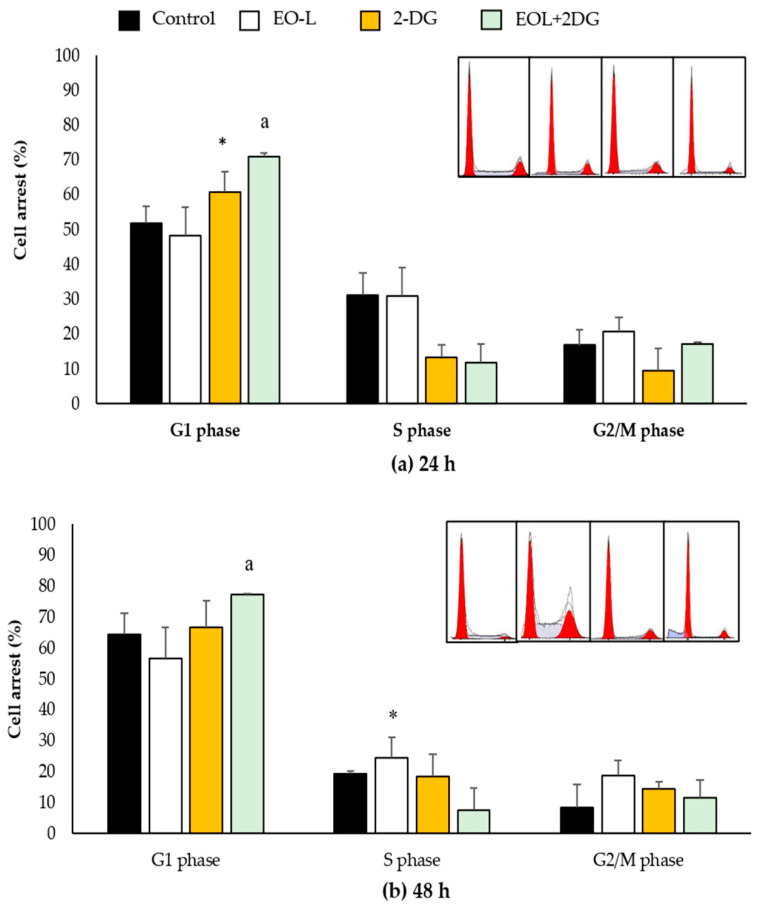

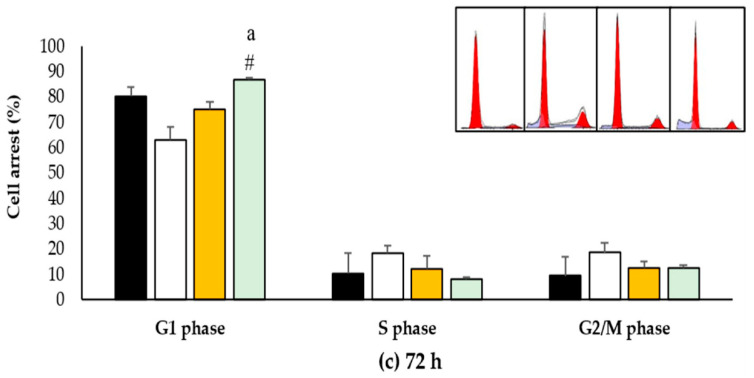
Induction of cell cycle arrest in PANC-1 cells. The percentages of cells in G1, S and G2/M phases were analyzed by flow cytometry after (**a**) 24 h, (**b**) 48 h and (**c**) 72 h. The data were shown as the mean values ± S.D. for three independent experiments, and the statistical analysis was determined using a Student’s *t*-test, with the * symbol showing a significant difference (*p* < 0.05) in comparison to control untreated cells, the # symbol showing a significant difference (*p* < 0.05) in comparison to 2-DG and the a symbol showing a significant difference (*p* < 0.05) in comparison to EO-L.

At 24 h (Figure 12a), the percentages of control cells (untreated) in the G1, S and G2/M phases were 51.99%, 31.15% and 16.8%, respectively. EO-L slightly decreased the percentage of cells in the G1 phase (48.36%), slightly increased the percentage of cells in the G2//M phase (20.74%), but there was no apparent change in the percentage of cells in the S phase (30.89%) when compared to control (untreated cells). In the cells treated with 2-DG, a significant (*p* < 0.05) increase in the percentage of cells in the G1 phase (60.77%) was observed, followed by a decrease in the S and G2/M phases by 13.31% and 9.61%, respectively. EOL+2DG treatment was also found to significantly (*p* < 0.05) increase in the percentage of cells in the G1 phase (70.93%) of the cell cycle, with a decrease in the S phase (11.81%) and no apparent effect in the G2/M phase (17.25%) compared to the control.

At 48 h (Figure 12b), the percentages of control cells in the G1, S and G2/M phases were 64.47%, 19.35% and 8.53%, respectively. In the cells treated with EO-L, significant (*p* < 0.05) increases in the percentages of cells at the S and G2/M phases were recorded at 24.5% and 18.74%, respectively. A slight increase in the percentage of cells in the G2/M phase after 48 h incubation with 2-DG was shown to be 14.38%, as compared to the control cells at 8.53%. The EOL+2DG treatment consistently induced cell cycle arrest at the G1 phase by 77.49% (*p* < 0.05). 

At 72 h (Figure 12c), similar findings were demonstrated in PANC-1 cells treated with EO-L, 2-DG and EOL+2DG. EO-L caused slight increase in the percentages of cells in the S and G2/M phases, as compared to the control. A similar observation was shown at 48 h, as 2-DG caused a slight increase in the accumulation of cells at the G2/M phase when compared to the control. Meanwhile, EOL+2DG significantly induced cell cycle arrest at the G1 phase after 72 h of treatment.

### 2.8. Effects of EO-L, 2-DG and EOL+2DG on P53, HIF1α, HK2 and CYP3A5 mRNA Expression in PANC-1 Cells

**Figure 13:**
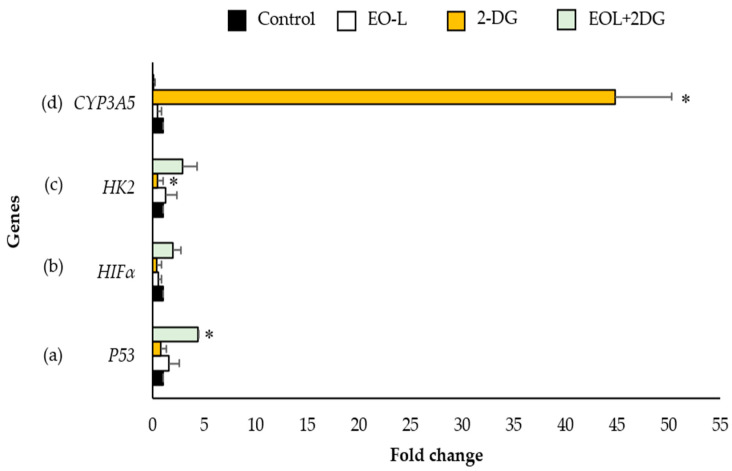
mRNA expression of (**a**) P53, (**b**) HIFα, (**c**) HK2 and (**d**) CYP3A5 in PANC-1 cells treated with EO-L, 2-DG and EOL+2DG. The fold change was calculated as a relative change in each mRNA level in comparison with the control expression level (expression of equals to 1.0). The mean values ± S.D. were obtained from three independent experiments that ran in triplicate. Statistical significance was assigned to a fold change based on a Student’s *t*-test between control (untreated cells) and treatment samples expression values, with the * symbol showing a significant difference (*p* < 0.05) in comparison to control cells.

In this study, quantitative polymerase chain reaction (qPCR) was performed to quantify the level of mRNA expression of P53, HIF1α, HK2 and CYP3A5 between each treatment in comparison to the untreated cells (negative control). The relative changes in mRNA expression were analyzed using the comparative ΔΔCt formula [24] and represented by the values of fold change (Figure 13a–d). Beta-actin (β-actin) was used as a housekeeping gene and an internal control to normalize the gene expression value of the target gene.

Figure 13a shows the mRNA expression of P53 in PANC-1 cells. The results showed that the mRNA levels of tumor suppressor gene P53 was 1.58-fold in EO-L treated cells in comparison with the control. In cells treated with 2-DG, the level of P53 was found to be slightly suppressed by 0.78-fold in comparison with the control. The EOL+2DG treatment significantly increased the mRNA expression of P53 4.40-fold (*p* < 0.05).

The expression level of H1F1α gene in PANC-1 cells treated with EO-L and 2-DG for 24 h decreased by 0.52 and 0.37-fold, respectively, as compared to the expression level in untreated control cells. It was found that the expression level of H1F1α was increased 1.92-fold after treatment with EO-L+2DG, as compared to the control (Figure 13b).

The expression level of HK2 was differently modulated by EO-L, 2-DG and EO-L+2DG, as evidenced by the measured expression levels being 1.25-fold, 0.45-fold (*p* < 0.05) and 2.88-fold, respectively, as compared to the levels in untreated control cells (Figure 13c).

Similarly, the expression level of CYP3A5 was modulated differently by EO-L, 2-DG and EO-L+2DG. The expression levels of this gene were significantly decreased 0.47-fold and 0.11-fold after the treatment with EO-L and EO-L+2DG, respectively, as compared to the control. On the contrary, the expression level of CYP3A5 was significantly increased 44.74-fold (*p* < 0.05) after treatment with 2-DG compared to the control cells (Figure 13d).

## 3. Discussion

This study focuses on the cellular and molecular mechanism of EO-L and its efficacy when combined with 2-DG, a known glucose metabolism inhibitor. This is the first study that provides evidence on the mechanistic activities possessed by the combination treatment between EO-L and 2-DG on the PANC-1 cell line. It has been mentioned that the findings based on whole-genome exome sequencing found 12 core signaling pathways and processes whose component genes were genetically altered in most pancreatic cancers. The 12 core signaling pathways include apoptosis, control of G1/S phase transition, Hedgehog signaling, Wnt/Notch signaling, chromatin regulation, integrin signaling, JNK signaling, KRAS signaling, TGFβ signaling, homophilic cell adhesion, invasion, small GTPase signaling and DNA damage control [25]. 

The pantropical genus *Garcinia* is the largest genus that belongs to the family Clusiaceae and comprises more than 400 species from all over the world [21]. The genus has received the attention of pharmaceutical industries for its edible fruits and nutraceutical properties. The Malaysian region is known to be the center of diversity of *Garcinia* species [21]. The well-known species, *Garcinia atroviridis* (*G. atroviridis*), is widely used as a flavoring cuisine by the Malay community. Moreover, it has been exploited by the international industry as a health food that can burn highly potent fats, as well as lower cholesterol, hypertension and rheumatism [19]. The plant has proven to exhibit several potential therapeutic activities, possibly attributable to its mixture of bioactive phytochemical contents. These activities were supported by various bioassay-guided studies that demonstrated the potential for anti-microbial [26], antihyperlipidemic [27], antioxidant [28], anti-inflammatory [29] and cytotoxic activities [22]. 

Essential oils can be defined as concentrated natural plant products which contain a mixture of volatile compounds, mainly mono- and sesquiterpenoids, benzoids, and phenylpropanoids. Various studies reporting on the uses, effects and modes of action of essential oils extracted from genus *Garcinia* have been previously published [13,22,26,27,28,29]. Regarding the phytochemical analysis of essential oils extracted from *G. atroviridis*, we recently reported that the oils from the leaves (EO-L) of this plant were found to contain 64% (E)-β-farnesene and 19% β-caryophyllene [22]. This preliminary study demonstrated the potential cytotoxic effect of EO-L to induce 50% cell death in a human breast cancer cell line (MCF-7) at a concentration of 71 µg/mL. In the present study, the efficacy of EO-L was further evaluated in the growth of a human pancreatic cancer cell line, namely PANC-1. The results of this study clearly demonstrate that EO-L potently possesses anti-proliferative effects against PANC-1 cells in a concentration- and time-dependent manner. The calculated constant IC_50_ of EO-L in PANC-1 cell line was 78 µg/mL. In this study, PANC-1 cells treated with EO-L showed a significant amount of LDH released from as early as 24 h in the incubation time. The increase in LDH leakage indicated that the plasma membrane of PANC-1 cells was damaged, thus supporting that EO-L is cytotoxic to PANC-1 cells. 

Owing to the fact that 2-DG causes cancer cell death through its effect by glucose-deprivation, the combination of treatments between EO-L at its sub-toxic concentration (IC_50_) led to higher 2-DG-induced cytotoxic and apoptotic effects in PANC-1 cells. In this study, the anti-proliferative effects between EO-L and 2-DG were further analyzed by calculating the values of the combination index (CI). CI is a value that quantitatively illustrates the interaction or relationship between the combination of two or more treatments. According to Chou [30,31], the CI values of less than one, equal to one and more than one represent synergism, additivity and antagonism, respectively. The combination between EO-L and 2-DG (EOL+2DG) demonstrated synergism and moderate synergism, and these were dependent on the concentrations of 2-DG and incubation periods. Critically, this study is the first to provide bioassay-guided mathematical evidence that showed the combination of EOL+2DG is more potent than the effects of 2-DG alone in inhibiting the growth of PANC-1 cells.

In contrast to normal cells, cancer cells are known to be under constant stresses, including aberrant cell growth and proliferation, oncogenic stress, genomic instability and cellular hypoxia [32]. Besides that, cancerous cells also show a reduced rate of apoptotic cell death [33]. Apoptosis is a programmed cell death that is normally activated to eliminate potential cancer cells. For this reason, apoptosis is conceivable as a positive process that prevents and treats cancer. Normally, the intrinsic pathway of apoptosis is activated in response to numerous stimuli. Mitochondria play major roles in sustaining cell survival by converting nutrients into energy (ATP) via oxidative phosphorylation [34]. This function is facilitated by the distinct characteristic of the inner mitochondrial membrane that it is known to be highly impermeable. This characteristic is important to generate the electrochemical potential necessary for oxidative phosphorylation and ATP production [35]. It is worth noting that the mitochondria of cancer cells display higher mitochondrial membrane potential (MMP). In addition, the depolarization in the structure of this organelle could activate the mechanism of apoptosis intrinsically [36]. The integrity of MMP could be tracked by using a cationic dye JC-1 assay [37]. In principle, the fluorescent dye JC-1 enters the mitochondrial matrix and stains the mitochondria of healthy cells red due to the formation of J-aggregates. On the contrary, apoptotic cells are stained green by JC-1 dye, as the dye molecules accumulate in the cytoplasm due to the collapse of mitochondria membrane integrity [38]. In the present study, both the EO-L and EOL+2DG treatments resulted in the prominent depolarization of MMP, as qualitatively and quantitatively determined by the reactivity with JC-1.

Apoptosis and programmed necrosis are the main forms of programmed cell death and can be distinguished by their morphological differences. The morphological changes in apoptotic cells include phosphatidylserine exposure on the outer surface prior to membrane damage [39]. These apoptotic cells were detected in the current study by staining the cells with a fluorescence-conjugated Annexin-V antibody that binds to phosphatidylserine. Propidium iodide (PI) has been used in this study to discriminate between apoptotic and necrotic cell death, in which the cells with PI-positive staining were regarded as undergoing necrosis. Flow cytometric analysis showed that EO-L induced the deaths of PANC-1 pancreatic cancer cells by apoptosis. 

*P53* is one of the key players in promoting apoptotic signals that are received by the mitochondria in the intrinsic pathway of apoptosis, and this defines the function of *P53* as a pro-apoptotic factor and tumor inhibitor [40]. Tumor protein 53, commonly abbreviated as *TP53* or *P53*, is a gene that codes for a protein involved in regulating the cell cycle. Due to this function, *P53* is commonly regarded as the ‘guardian of the genome’, as it regulates genome stability. It plays a major role in modulating cellular response to cytotoxic stresses by contributing to cell cycle arrest and programmed cell death. First, it controls the cell cycle delay, which is accompanied by the repair of DNA damage. Otherwise, *P53* exerts its tumor suppressor function by directing the cells to undergo apoptosis or by permanent cell cycle arrest through termination by necrosis or cell differentiation. *P53* was shown to mediate the arrest of cells at the G1 to S boundary, G2 and the mitotic checkpoints. This allows the cells to repair the DNA damage prior to the synthesis or replication of DNA and cell division. The cells that still retain the ‘irreparable’ or high DNA damage will be subsequently navigated to apoptosis. Therefore, the loss of *P53* function during carcinogenesis can lead to aberrant cell growth, increased cell survival of damaged cells and genetic instability. In pancreatic cancer, the *P53* tumor suppressor gene is inactivated in approximately 50–75% of the cases, with the most frequent mechanism of inactivation being genetic mutation with the loss of second allele [5,25]. This illustrates that the two critical controls of cell number (cell division and cell death) are deregulated in the majority of pancreatic cancers. 

In the present study, EO-L caused a slight increase of cells accumulated at the S and G2/M phases. The mode of action induced by 2-DG varies to a certain extent in regulating the cell cycle in PANC-1, depending on the incubation period. In this study, 2-DG was found to increase the accumulation of PANC-1 cell in the G1 phase at 24 h, and in the G2/M phase after 48 and 72 h of incubation periods. On the contrary, EOL+2DG consistently induced significant G1 phase arrest of PANC-1 cells throughout all the incubation periods tested in this study. Molecular biology data acquired by real-time PCR further supported the aforementioned cell cycle arrest. The expression of *P53* mRNA in PANC-1 cells was significantly increased after EOL+2DG treatment. Taken together, these findings suggested that the *P53* activation may be partly involved in cell cycle arrest and apoptotic cell death of PANC-1 cells treated with EO-L+2-DG. The present study also hypothesizes that the G1 arrest and apoptosis triggered by EOL+2DG are likely mediated by coordinated induction of the *P53*-dependent pathway. However, the induction of apoptosis by EO-L treatment was not involved with *P53* signaling. This finding could postulate that EO-L induces apoptosis in a *P53*-independent pathway. Nevertheless, a further experiment should be carried out in the future to validate the aforementioned hypothesis.

*HIF-1α* is commonly overexpressed in pancreatic cancer, and it is further linked to greater cancer invasion and metastasis. *HIF-1α* is the main regulator of cell response towards oxygen concentration [41], thus enabling PDAC cells to survive in an oxygen-deficient environment. In addition, *HIF-1α* also plays an essential role in regulating the cellular metabolism and the increasing anti-apoptotic capacity of pancreatic cancer cells [42]. Besides, one study on pancreatic cancer cell demonstrated that the *HIF-1α* gene might also act as a tumor suppressor by preventing the expression of *PPP1R1B* by activating the *P53* gene that results in cancer cell death. Furthermore, it has been demonstrated that the knockout of *HIF-1α* gene in pancreatic cancer cells may result in the more invasive form of cancerous cell growth [43].

Chiavarina and colleagues [44] reported the dual functions of *HIF-1α* and demonstrated that the *HIF-1α* functions as a tumor promoter in cancer-associated fibroblasts, as well as a tumor suppressor in MDA-MB-231 breast cancer cells. Based on their findings, the authors concluded that the functions of *HIF-1α* were determined by the types of cells. The present study demonstrated that EO-L and 2-DG slightly reduced *HIF-1α* level, while EOL+2DG slightly increased *HIF-1α*. Therefore, the findings obtained from this study indicated that the types of treatments might affect the expression and function of *HIF-1α* in PANC-1 cells. EO-L, 2-DG and EOL+2DG treatments differently modulated the expression of *HIF-1α* but resulted in the death of PANC-1 cells. 

One of the hallmarks of cancer cells is accelerated glucose metabolism under aerobic conditions [45]. In this condition, also referred to as the Warburg Effect, glucose is converted to lactate in the presence of oxygen [46]. An increase in glucose metabolism is required to provide sufficient amounts of metabolic intermediates. These metabolic intermediates are required in rapidly dividing cancer cells for the synthesis of new nucleic acids, lipids and proteins. The function of hexokinases is to catalyze the first committed step of glucose metabolism [47]. For example, this enzyme accelerates the process of ATP-dependent phosphorylation in glucose molecules to produce glucose-6-phosphate (G6P). By catalyzing this process, it promotes and sustains a concentration gradient that facilitates glucose entry into the cells [48]. *HK2,* one example of hexokinases isoforms, is highly expressed in cancer cells. This gene, at least in part, is responsible for the accelerated glucose flux and important in cancer cell survival [49]. In the present study, 2-DG significantly reduced the expression of *HK2*. This was supported by a previous study that also found that 2-DG was able to inhibit prostate cancer cell proliferation by inducing cell apoptosis and autophagy by targeting the *HK2* gene [50]. On the contrary, the expression of *HK2* was increased in cells treated with EO-L and EOL+2DG. These results suggest that the anti-proliferative effects of EO-L and EOL+2DG are independent of *HK2* activation. 

The cytochrome P450 genes encode enzymes that are involved in the formation (synthesis) and breakdown (metabolism) of various molecules and chemicals within the cells. In the present study, one member of cytochrome P450 superfamily genes, namely *CYP3A5,* was used to evaluate the effects of EO-L, 2-DG and EOL+2DG. Accordingly, the *CYP3A5* gene is a part of the cytochrome P450 gene group, clustered under group 3, subgroup A and associated with gene 5 [51]. 

In the present study, the expression of *CYP3A5* gene was significantly up-regulated by the treatment of 2-DG, while the treatments of EO-L and EOL+2-DG showed no significant modulatory effects on this gene in PANC-1 cells when compared to the control cells. This finding may suggest that the genetic variations in *CYP3A5* metabolism should be considered upon the treatments with EO-L, 2-DG or EOL+2DG. *CYP3A5* is known to participate in metabolizing actions of drugs and foreign chemicals [52,53]. Furthermore, its *CYP450* superfamily gene products (enzymes) were reported to cause clinically significant drug interactions [54,55]. The authors also described that an alteration of *CYP450* metabolism results in variations in drug interactions and responses. A drug is referred to as an enzyme inhibitor if it blocks the metabolic activities of one or more CYP450 enzymes [55]. This, in turn, will slow down the substrate drug metabolism and increases the drug’s effect. This may indicate that the drug may stay active longer, so less concentration is needed in order to attain the desired effect. In contrast, a drug is referred to as an enzyme inducer if it increases CYP450 enzyme activity by increasing enzyme synthesis [55]. This, in turn, will speed up the substrate drug metabolism and decreases the drug’s effect. This may indicate that a higher dose might be needed in order for the drug to be effective because it is quickly metabolized and broken down. In this present study, it can be suggested that 2-DG was a potent enzyme inducer in the PANC-1 cell line, whereas neither EO-L nor EOL+2DG could be considered as potent enzyme inducers or enzyme inhibitors. EO-L and EOL+2DG may target different expressions of the CYP450 family. Despite that, the information regarding a drug’s CYP450 metabolism and its potential for inhibition or induction should be taken into consideration, as it may affect pharmacokinetics and pharmacodynamics in the human body.

## 4. Materials and Methods

### 4.1. Cell Culture

Human pancreatic cancer cell line PANC-1 was obtained from AddexBio Technologies (San Diego, CA, USA) and lung normal cell line BEAS-2B was kindly provided by Associate Professor Dr Badrul Yahaya from Cluster of Regenerative Medicine (AMDI, USM). PANC-1 and BEAS-2B cells were, respectively, cultured in DMEM (Grand Island Biological Company, Waltham, MA, USA) and DMEM/F12 (GIBCO BRL, Thermo Fisher Scientific, Waltham, MA, USA) media containing 10% (*v/v*) FBS (GIBCO BRL, USA) and 0.1% (*v/v*) penicillin-streptomycin (GIBCO BRL, USA). Both of the cells were maintained in a humidified atmosphere with 5% CO_2_ at 37 °C. 

### 4.2. Preparation of G. atroviridis Essential Oil (EO-L) and 2-Deoxy-d-glucose (2-DG)

Around 95 g of *G. atroviridis* leaves were used for essential oil extraction. Firstly, the leaves were subjected to hydrodistillation process for 5 h by using a Clevenger-type apparatus and with distilled *n*-pentane as the collecting solvent. The resulting oil (EO-L) was concentrated under a gentle flow of nitrogen gas. Then, the concentrated resulting oils from *G. atroviridis* leaves (EO-L) were dissolved in dimethyl sulphoxide (DMSO) at a stock concentration of 10 mg/mL and kept at −20 °C until further use. 

The 2-DG powder was purchased from Nacalai Tesque (Kyoto, Japan). The 2-DG stock of 10 mM was prepared by diluting 15 mg of 2-DG powder with 9.1 mL of sterile distilled water. Then, the solution was filtered using a 0.22 µM syringe filter and kept in −20 °C until further use.

### 4.3. Cell Proliferation Assay

PANC-1 cells were seeded at 5 × 10^3^ cells/well, while BEAS-2B cells were seeded at a concentration of 1 × 10^4^ cells/well in the 96-well plates. Next, the plates were left overnight in a 5% CO_2_ incubator at 37 °C. After that, the cells were treated with EO-L at different concentrations ranging from 10 to 100 µg/mL for 24, 48 and 72 h. In addition, the cells were treated with 2-DG as a positive control at different concentrations ranging from 1 to 4 mM. Similarly, the plates were left overnight in a 5% CO_2_ incubator at 37 °C. After the treatment, the cells were stained with 10 µL (5 mg/mL) of MTT solution (Merck Millipore, Darmstadt, Germany). The optical density (O.D) of each well was measured at 570 nm, with a reference wavelength of 620 nm, using a microplate reader (PowerWaveXS, Bio-Tek, Winooski, VE, USA). Cell viability was determined using formula:

[(O.D treatment − O.D blank)/(O.D untreated cell − O.D blank)] × 100%(2)

### 4.4. Cytotoxicity (LDH-Release) Assay

PANC-1 cells were seeded in 24-well plates (SPL Lifesciences, Gyeonggi-go, Korea) at a concentration of 1 × 10^5^ cells per mL, and were allowed to attach overnight. The process was followed by the treatments with EO-L (10–100μg/mL) and 2-DG (1–4 mM), respectively, for up to 72 h at 37 °C and with 5% CO_2_. The control cells were treated with the vehicle, culture medium. Cytotoxicity effect of EO-L on PANC-1 was determined by using lactate dehydrogenase (LDH)-release assay (Roche Diagnostics, Mannheim, Germany), as described by the manufacturer’s protocol. The optical density (O.D) of each well was measured at 490 nm, with a reference wavelength of 620 nm using a microplate reader (BIO-TEK PowerwaveXs). Cytotoxicity was determined using the formula stated below:

% Cytotoxicity = (Treated cells − Low control)/(High control − Low control) × 100(3)

### 4.5. Selective Index (SI) Analysis

The selectivity index (SI) of a cytotoxic agent was calculated by obtaining the ratio of IC_50_ in the non-cancerous cell line, as compared to the IC_50_ in the cancerous cell line. In this study, the SI of EO-L was calculated by using the formula stated below:

IC_50_ BEAS-2B cells/IC_50_ PANC-1 cells (4)

### 4.6. Combination Index (CI) Analysis

The effects of combinational treatment between EO-L and 2-DG on PANC-1 cells were assessed by using an MTT assay (Merck Millipore, Darmstadt, Germany). The cells were treated with EO-L (IC_50_ value) in combination with 2-DG at 1, 2, 3 and 4 mM, respectively, followed by incubation for 24 to 72 h. The combination index (CI) values were calculated by using CompuSyn software version 1.0 (ComboSyn Inc., Paramus, NJ, USA). The CI value was indicated with less, equal and more than 1 indicate synergism, additivity and antagonism, respectively. 

### 4.7. Assessment of Mitochondrial Membrane Potential (MMP)

The MMP changes in PANC-1 cells were quantitatively and qualitatively measured by staining the cells with JC-1 cationic dye (Cayman Chemical, An Arbor, MI, USA) for 24, 48, and 72 h duration, following the manufacturer’s protocol. Briefly, after each time point, 10 µL of JC-1 dye was added into each well, and the cells were incubated for 30 min. Following that, the cells were washed with 1× cold assay buffer. The stained cells were then analyzed by using a fluorescence plate reader (FLUOstar Omega, Ortenberg, Germany) and fluorescence microscope (Olympus, Center Valley, PE, USA).

### 4.8. Assessment of Mechanism of Cell Death (Apoptosis and Necrosis)

Apoptotic cells were measured using an Annexin V FitC Apoptosis Detection Kit (Becton Dickinson Biosciences, Franklin Lakes, NJ, USA). PANC-1 cells were seeded at 1 × 10^5^ cell concentration and treated accordingly. The treated cells were washed twice in 1X cold phosphate buffered saline (PBS) (BD Biosciences, USA), centrifuged and then the cell concentration was adjusted at 1 × 10^6^ cells/mL. After that, 100 µL of cells were transferred to an empty culture tube. The cells were double-stained with 5 µL of Annexin V FitC (BD Biosciences, USA) and propidium iodide (BD Biosciences, USA), followed by incubation in cold and dark conditions for 15 min. Finally, 400 µL of the binding solution was added and analyzed using flow cytometry (Becton Dickinson FacsCalibur, Franklin Lakes, NJ, USA).

### 4.9. Analysis of Cell Cycle Distribution

Cell cycle distribution was measured using a Cycle TEST™ PLUS DNA Reagent Kit (BD Biosciences, Franklin Lakes, NJ, USA) and following the manufacturer’s protocol. PANC-1 cells were seeded at a concentration of 1 × 10^5^ cells/mL and left overnight in an incubator. Prior to the treatment, the cells were serum-starved for 24 h and were treated accordingly the next day. Briefly, the floating and adherent cells were collected, spun and washed with 1X cold PBS. Then, the concentrations of cells were adjusted to 1 × 10^6^ cells/mL and stained with propidium iodide (BD Biosciences) in the presence of RNAse buffer (BD Biosciences) for 30 min on ice and in a dark condition. The results of staining with 15,000 cells per sample were then collected by flow cytometry (Becton Dickinson FacsCalibur, Franklin Lakes, NJ, USA) and analyzed using Modfit Lt 5.0 software (Becton Dickinson).

### 4.10. Quantitative Measurement of mRNA Expression by Real Time Polymerase Chain Reaction (RT PCR)

PANC-1 cells were seeded in t-25 flasks at a density of 1 × 10^6^ cells (4 mL/flask) for 24 h. The cells were collected following treatment with EO-L, 2-DG and EOL+2DG for 24 h, respectively. Total RNA was extracted from the collected cells using a TanszolUp Plus RNA kit (TransGen Biotech Limited Company, Beijing, China) according to the manufacturer’s protocol. A Transcript Green One-Step qRT-PCR Supermix kit (Transgen Biotech Limited Company, Beijing, China) was used to transcribe the total RNA into cDNA, according to the manufacturer’s protocol. qPCR was performed with an ABI Step One Plus (Thermo Fisher Scientific, Waltham, MA, USA) real-time PCR system. The PCR process was initiated at 45 °C for 5 min, followed by 40 cycles at 94 °C for 35 s and 60 °C for 30 s. The primers used are as listed in the Table 2 below:

### 4.11. Statistical Analysis

The data were acquired from at least three independent experiments. Statistical analysis on the collected data was performed by using the Student’s independent *t*-test in SPSS software (International Business Machine^®^ Statistical Package for the Social Sciences^®^ Statistics, Version 24, Chicago, IL, USA). The value of *p* < 0.05 was considered significant when compared to control (untreated cells).

## 5. Conclusions

In conclusion, this study demonstrates the potential of EO-L from *G. atroviridis* in inhibiting the growth of PANC-1 cells through the actions of increasing the release of LDH and MMP, in addition to causing apoptosis and cell cycle arrest at the S phase. This is despite the insignificant results measured from the mRNA expression analyses in *P53*, *HIFα*, *HK2* and *CYP3A5* genes. Most importantly, this study found that EO-L was able to synergize the actions of 2-DG in promoting PANC-1 cell death in vitro.

## Figures and Tables

**Table 1:** Combination index (CI) values of EOL+2DG treatments at 24, 48 and 72 h.

24 h				
EO-L (µg/mL)	2-DG (mM)	Fraction Affected (fa)	CI	Degree of Synergism/Graded Symbols
78	1	0.17	0.73 ± 3.65	Moderate synergism (++)
78	2	0.46	0.348 ± 9.29	Synergism (+++)
78	3	0.57	0.368 ± 5.21	Synergism (+++)
78	4	0.59	0.405 ± 1.26	Synergism (+++)
48 h				
78	1	0.36	0.892 ± 3.96	Moderate synergism (++)
78	2	0.55	0.582 ± 7.07	Synergism (+++)
78	3	0.67	0.431 ± 7.21	Synergism (+++)
78	4	0.62	0.757 ± 4.80	Moderate synergism (++)
72 h				
78	1	0.81	0.741 ± 4.55	Moderate synergism (++)
78	2	0.84	0.595 ± 0.26	Synergism (+++)
78	3	0.88	0.457 ± 2.49	Synergism (+++)
78	4	0.87	0.518 ± 1.12	Synergism (+++)

**Table 2:** List of primer sequences.

List of Primers	Primer Sequences	
*β actin*	Forward	5′-GTGGGAGTGGGTGGAGGC-3’
	Reverse	5′-TCAACTGGTCTCAAGTCAGTG-3’
*P53*	Forward	5′-GAGCTGAATGAGGCCTTGGA-3’
	Reverse	5’-CTGAGTCAGGCC CTTCTGTCT T-3’
*HIF1α*	Forward	5′-CATAAAGTCTGCAACATGGAAGGT-3’
	Reverse	5′-ATTTGATGGGTGAGGAATGGGTT-3’
*HK2*	Forward	5’-CAAAGTGACAGTGGGTGTGG-3’
	Reverse	5′-GCCAGGTCCTTCACTGTCTC-3’
*CYP3A5*	Forward	5′-CTATCGTCAGGGTCTCTGGAAATT-3’
	Reverse	5′-ACGTTCCCCACATTTTTCCATA-3’

The relative levels of four targeted genes were calculated with the comparative 2^−^^ΔΔCt^ method.

## Data Availability

Not Applicable.

## Sample Availability

Not available.
